# Intrahospital and Territorial Management of Violence Against Children in the Verbano-Cusio-Ossola Area, Northern Italy

**DOI:** 10.3390/ijerph23020223

**Published:** 2026-02-10

**Authors:** Giulia Riva, Elena Rubini, Mattia Mazzola, Antonella Tedesco, Lorenza Scotti, Sarah Gino

**Affiliations:** 1 School of Medicine, University of Eastern Piedmont, 28100 Novara, Italy; 20013052@studenti.uniupo.it; 2 Department of Public Health, Experimental and Forensic Medicine, University of Pavia, 27100 Pavia, Italy; 3 CRIMEDIM—Center for Research and Training in Disaster Medicine, Humanitarian Aid and Global Health, University of Eastern Piedmont, 28100 Novara, Italy; elena.rubini@uniupo.it; 4 Department for Sustainable Development and Ecological Transition, Università del Piemonte Orientale, 13100 Vercelli, Italy; 5 SOC Neuropsichiatria Infantile—ASL VCO, 28922 Verbania, Italy; mattia.mazzola@aslvco.it; 6 SOS Direzione Sanitaria Presidio Ospedaliero di Verbania, 28922 Verbania, Italy; antonella.tedesco@aslvco.it; 7 Department of Translational Medicine, University of Eastern Piedmont, 28100 Novara, Italy; lorenza.scotti@uniupo.it; 8 Department of Health Sciences, University of Eastern Piedmont, 28100 Novara, Italy

**Keywords:** child abuse, child maltreatment, risk factors, secondary prevention, emergency department, territorial network

## Abstract

**Highlights:**

**Public health relevance—How does this work relate to a public health issue?**

- Under-reported crisis: Child maltreatment is a major global public health issue that is often overlooked.
- Multidimensional violence: It encompasses physical, sexual and psychological abuse, as well as neglect and exposure to domestic violence.

**Public health significance—Why is this work of significance to public health?**

- Diagnostic gap: There is a critical disconnect between hospital settings and territorial networks, with many cases of abuse being missed in emergency departments.
- Transgenerational risk: Caregivers often have a history of adverse childhood experiences (72%) or psychiatric comorbidities (27%), perpetuating a cycle of violence.

**Public health implications—What are the key implications or messages for practitioners, policy makers and/or researchers in public health?**

- Standardized protocols: Implementing specific diagnostic codes and universal guidelines in hospitals is vital for accurate identification.
- Specialized training: Healthcare providers require targeted education to recognize “indicators of suspicion” and coordinate with multidisciplinary teams.

**Abstract:**

Violence against minors remains a significant, often under-reported public health crisis. This study evaluated the incidence and clinical management of child abuse within the Verbano-Cusio-Ossola area (Northwest Italy) from January 2017 to August 2023. A retrospective descriptive analysis was conducted using two primary data streams: (1) Territorial data: records from the multidisciplinary “Maltrattamento, Trascuratezza, Abuso” (“Maltreatment, Neglect, Abuse”) team. (2) Hospital data: Pediatric Emergency Department admissions in Verbania and Domodossola, screened via diagnostic filters and ICD-related codes. At the territorial level, 161 minors were identified. While the territorial network demonstrated high activation rates (96.25%) and legal reporting (92.55%), a history of missed reports was noted in 8.13% of cases. Parental risk factors were prevalent: 72% of caregivers reported adverse childhood experiences and 27% presented with psychiatric comorbidities. In contrast, hospital data revealed a diagnostic gap. Out of 1,586 pediatric ED admissions, only one case was explicitly recorded as child abuse. Furthermore, none of the discharges utilized specific maltreatment diagnostic codes, despite several patients presenting with recurrent “accidental” traumas. These findings highlight a disconnect between community services and acute clinical settings. Enhancing intrahospital surveillance and implementing specialized training for healthcare providers are essential to bridge this diagnostic gap and ensure a coordinated, multidisciplinary response to child maltreatment.

## 1. Introduction

The World Health Organization (WHO) defines violence against children as any kind of abuse against people under 18 years old which can cause damage to their health, survival, development, and dignity in the context of a trusting relationship [1]. This phenomenon is a global issue, with one out of every two people under the age of 18 experiencing some form of violence in 2020 [2]. Despite these alarming figures, this type of abuse is still an underestimated problem.

Child maltreatment encompasses physical, psychological, and sexual abuse as well as witnessed violence (e.g., when a minor witnesses violent acts perpetrated against an emotionally significant figure), neglect which also includes abuse connected to lack of, inappropriate, or excessive medical care, and peer violence.

The management of cases of violence against children and adolescents is complex, and the first stage should involve early detection of the abuse. The “indicators of suspicion” can serve as supportive tools for professionals in recognizing and documenting non-specific clinical manifestations of abuse. In the context of emergency care, the “Escape form”, a six-item checklist evaluating aspects such as the coherence of the story, consistency between anamnesis and physical examination, and the behavior of the accompanying person, can be used [3]. Understanding the main risk factors (e.g., unemployment, psychiatric pathologies, and trans-generational violence) related to the profile of the victim and the abuser is another useful tool [4]. Recognition of child maltreatment in hospital or at a territorial level must be followed by a report to social services, judicial authority, or law enforcement agencies.

Clinical examination in hospital settings should follow strict guidelines such as choosing a protected environment, encouraging spontaneous storytelling during the anamnesis, and performing a physical top-to-toe examination by focusing on one area at a time, describing the injuries, and taking photographs of the lesions [5].

It is important to emphasize that violence can cause serious consequences such as neurological damage as well as delays in psycho-motor development and could be a risk factor for the development of psychopathological disorders, pathological addictions, and intergenerational abuses in adulthood. Therefore, after determining the presence of abuse, it is necessary to establish a therapeutic plan including regular follow ups for mistreated children and their family.

Despite the growing awareness of child maltreatment, localized data remains essential to tailoring effective public health responses. This study addresses this gap by focusing on the epidemiological characterization of violence against children and adolescents in the Verbano-Cusio-Ossola area (Piedmont, Italy).

The primary objective is to quantify the prevalence of this phenomenon and to map its clinical trajectory within both hospital and territorial sociosanitary networks. Specifically, the research aims to:

- Analyze the distribution of abuse cases through a systematic review of the records of the Emergency Department (ED) and of territorial sociosanitary facilities.
- Identify clinical and demographic patterns that may serve as early epidemiological markers of risk.
- Evaluate the efficacy of current management protocols by identifying systemic criticalities and undocumented warning signs in medical records.

By integrating clinical data with sociosanitary outcomes, this study seeks to provide a robust epidemiological baseline to enhance early recognition and refine prevention strategies within the regional healthcare system.

## 2. Materials and Methods

### 2.1. Setting

Victims of child abuse may be intercepted at hospital level in primary care facilities, which, in Italy, are under the jurisdiction of Local Health Authorities (Aziende Sanitarie Locali, ASL), through access to the ED, as well as in pediatric clinics and wards, and through the complex operating structure of Child Neuropsychiatry working at a territorial level.

Access at hospital level is via the emergency department, with a care pathway similar to that used for cases of gender-based violence (GBV) against adults. In cases where the victim is under 18 and has not suffered sexual violence, management occurs at ED level after informed consent is obtained from parents or the legal guardian. Psychosocial assistance is requested, ex officio reporting to the authorities is carried out, and follow-up preparation is undertaken, including establishing whether the victim can be discharged safely or if protection procedures (Art. 403 Civil Code) or shelter activation need to be put in place. If the victim is under the age of 14 and has suffered sexual violence, the case is managed in the pediatric ward; otherwise, it is managed in the obstetrics and gynecology unit.

Data collected include the description of how the abuse occurred (including date and time, location, number of aggressors and relationship between victim and aggressor, type of violence, use of substances, and prior episodes of violence), top-to-toe physical examination as well as psychological examination, collection of biological evidence and of photographs of the lesions, as well as STI and toxicological screening.

For primary care facilities, there is a referral to the ED.

At the VCO territorial level, the Child Neuropsychiatry complex operating structure acts in collaboration with social services and specialist services from ASL with interventions aiming at protecting and supporting mistreated and abused children through the MTA team, as well as through evaluation and management of suspicious or confirmed cases ordered by the judicial authorities.

Given the complexity of the setting, a parallel analysis encompassing both backgrounds (e.g., intra-hospital and territorial) was conducted.

It is worth making a preliminary remark regarding the legislative framework in the Piedmont region, where the study was conducted. In this regard, it is worth noting that, in 2022, the Piedmont region created a three-year plan of action against GBV for the period 2022–2024. The Plan, implementing Regional Law 4/2016 of the Piedmont Region, promotes prevention and protection activities as well as the creation of policies to protect women, girls, children, and vulnerable families. Compared to the 2017–2020 Plan, the 2022–2024 Plan extends and strengthens these measures, in particular by broadening the scope of protection: not only adult women, but also minors, minors who witness violence, migrant women, and the family of the victims. It also introduces a more integrated and systematic approach that enables networking between social services, health services, anti-violence centers, shelters, and other actors in the third sector, with the aim of ensuring that responses are consistent, coordinated, and regional in scale. It provides for specific training for operators, recognizing that case management requires appropriate skills and a multidisciplinary approach [6].

### 2.2. Data Collection

The study period taken into consideration spans from 1 January 2017 to 31 August 2023.

In the present study “children” will be used as an umbrella term to refer to different categories of patients (e.g., newborns, toddlers, and adolescents), while the expressions “violence” and “abuse” will be used interchangeably to refer to the variety of violent and physical acts perpetrated against this population.

In the territorial setting, cases of patients under the age of 18, of both sexes, that were victims of any form of violence and taken in charge by the Multidisciplinary team “Maltreatment, Neglect, Abuse” (MTA) were included. All cases taken into charge by the MTA were taken into consideration for the analysis. A database was filled in by using an anonymized chronological list of patients, consulting medical records and reports, and utilizing the SMAiL platform (the acronym SMAiL means “Multidisciplinary Information System for Adolescence and Childhood”. It is a Web Application that allows operators to constantly update clinical-therapeutic information on patients and the services provided, thus creating a regional network of child neuropsychiatry and psychology services) for patients’ records and services provided. Data to be extracted was also informed by the findings of a series of semi-structured interviews with healthcare staff conducted by one member of the research team (GR). The database comprised 30 variables (Supplementary File S1) later grouped into macro-categories: characteristics of violence (the categorization of the different types of violence adheres to that applied in medical records, reports, and reported by interviewees in semi-structured interviews), demographic characteristics of the child, patient management and activation of territorial network, characteristics of the abusive or neglecting adults, pathological addictions of the abuser and the victim, the child’s medical history, advice sought, involvement of the judicial authority, post-caretaking measures, and follow-up.

In the hospital context, the ADT-PS software (the ADT-PS software is an information system for the administrative management of patients accessing ED, including the pediatric room) was employed, and for medical records for patients under the age of 18 accessing the pediatric ED in Verbania and Domodossola clinical record review was employed to retrieve documentation of cases regarding abuses against minors, including a search for keywords such as “trauma”, “falls”, “burns”, and “adjustment disorders with anxiety and depressed mood”. Subsequently, the number of admissions that were followed by a hospitalization in the pediatrics department and the number of admissions that resulted in a discharge were assessed.

Finally, a cross-check on cases managed by the MTA team for which there had been previous accesses to the ED was conducted, evaluating the number of accesses and the presence of a recurrent “pattern of diagnosis” associated with these cases.

The variables extracted from ED records were selected based on their clinical relevance to pediatric maltreatment and their potential to flag under-recognition.

### 2.3. Statistical Analysis

The characteristics of the patients in terms of violence and outcomes suffered are summarized using descriptive statistics. Categorical variables were reported as absolute frequencies and percentages, while numerical variables were expressed as median and first and third quartiles.

The chi-square test or Fisher’s exact test for categorical variables and Mann–Whitney test for numerical variables were employed to evaluate the relationship between patients’ socio-demographic and violence characteristics.

The relationship between these data constitutes part of the analyzed outcomes. Outcome variables included previous failed reporting, the prompt activation of the territorial network, the correlation between the type of violence and the activation of the territorial network, cases taken in charge by the MTA team describing unfavorable growing conditions in the adult/abuser, cases managed by the multidisciplinary team where the adult/abuser suffers from a psychiatric pathology, and the evaluation of positive outcome (e.g., an improvement in the clinical, psychological, and social condition of the patient, and, in the case of intrafamily violence of the entire family unit, an improvement in the minor’s situation compared to their condition prior to care was sometimes achieved through alternative placement, such as foster care or adoption, away from the family of origin).

## 3. Results

### 3.1. Territorial Level

#### 3.1.1. Study Period

**Figure 1:**
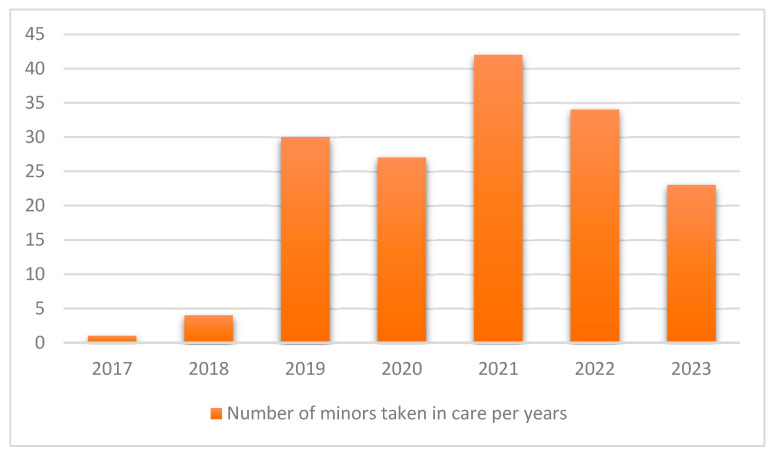
Number of minors taken into care at territorial level in the years covered by the study.

Between 1 January 2017 and 31 August 2023, 161 minors were taken into care at the territorial level. In 2019, the year in which the multidisciplinary team was formed, 30 patients were taken into care. During the first year of the COVID-19 pandemic (2020), there was a decrease in the number of patients taken into care (*n* = 27), significantly lower than the figure for the following year (2020), which represented a peak of 42 patients accepted. In 2023, only 23 minors were taken into care (Figure 1).

#### 3.1.2. Demographic Characteristics of the Victims

The median age of patients was 9.

Regarding the sex of victims, half of them were male.

Most patients were of Italian origin (77%); 19% were of non-European origin, with the remaining coming from Western Europe (e.g., Switzerland) and Eastern Europe (e.g., Albania and Romania). Most children were born in Verbania, the largest city in the area considered (57%), 35% of children or adolescents lived in small towns, 32% in tourist destinations and 22% in small cities. A significantly lower number of children lived in rural settings (a small town refers to municipalities with fewer than 5000 inhabitants; a small city refers to populated areas with between 5000 and 20,000 inhabitants (such as Omegna and Domodossola); a tourist city refers to locations with more than 20,000 inhabitants (such as Verbania)).

In 24% of cases, children attended primary school when violence occurred.

#### 3.1.3. Characteristics of Violence

Table 1 summarizes the kind of violence suffered by minors in the sample.

In almost half of the cases considered in the study, multiple episodes of abuse were found (66 out of 161, 41%).

The most frequently reported form of abuse was witnessed violence, occurring in 54% of cases (*n* = 87). For 98% (*n* = 85) of patients the perpetrators of witnessed violence were intrafamilial.

Concerning the relationship between victim and perpetrator, in 155 out of the entirety of cases (96%), the abuser belonged to the family unit, while extrafamiliar violence occurred only in 4 cases (2%).

The abuse mostly took place at home (90%) and only 2% of the cases occurred in a public place.

The number of cases involving prolonged violence (with the cut-off set at one month) was also investigated, identifying a protracted abuse in 148 cases (90%).

#### 3.1.4. Taking Charge of Minors and Activation of the Territorial Network

During data collection, particular attention was given to how the multidisciplinary team and the territorial services managed cases of child abuse. The number of cases detected, the frequency in the activation of the dedicated pathway, and the duration of intake were assessed.

A crucial element in the prevention of revictimization is the reporting to the judicial authorities performed by educational institutions, families, and health professionals. Therefore, patients’ conditions and risk factors related to the characteristics of the abusers were analyzed in order to understand how many cases could have been detected at an earlier stage. A missed detection of child abuse was found in 8% of the cases. The territorial network was promptly activated in 96% of cases.

#### 3.1.5. Characteristics of the Neglectful or Abusive Adult

Table 2 explores the characteristics of adult perpetrators of child abuse. Specifically, two factors that appear to have contributed to the early detection of conditions leading to the development of abusive and neglecting behaviors against children in perpetrators included in the study were highlighted, namely unfavorable growing conditions and the presence of psychiatric pathology or psychological weakness that may affect the parent–child relationship. Adults who were victims of violence during childhood were the perpetrators in 72% of cases, while adults with a diagnosed psychiatric condition made up 27% of cases. The number of individuals experiencing psychological weakness was higher at 47%.

#### 3.1.6. Pathological Addictions

Adult perpetrators suffering from pathological addictions at the time of intake accounted for almost half of the cases (46%). In contrast, the percentage of victims who had taken substances or alcohol at the time of abuse was lower, at 5%.

#### 3.1.7. Child’s Medical History

The presence or absence of symptoms or signs manifested by the child following the episodes of violence, as well as their type, were investigated to understand the most frequent consequences of abuse or maltreatment. In 85% of cases, the child developed sequelae due to the violence suffered. The most frequently detected outcomes were affective disorders (e.g., major depression, persistent depressive disorder, disruptive mood dysregulation disorder, manic episode, and hypomanic episode).

#### 3.1.8. Advice Sought

The type of advice required by the multidisciplinary team was evaluated to understand the degree of interaction between the involved professionals. The most requested consultation was “child neuropsychiatry” in 21 out 161 cases (19%). Medical–legal counseling (e.g., in the ED) was reported in 2% of cases. Requests for counseling prior to charge-taking by the MTA team were not considered, as at the operational level the intervention of the team in a territorial context is not coded as an emergency.

#### 3.1.9. Involvement of the Judicial Authority at Territorial Level

In 92% of cases a complaint was made to the judicial authority.

The distribution of types of violence stratified by presence or absence of the judicial authority’s involvement is reported in Table 3.

#### 3.1.10. Post-Taking Care Measures and Follow-Up

In 68% of the cases, measures were put in place to protect the child. The primary action taken was the removal of the perpetrator from the abused person in 64% of the cases.

Moreover, for children in the sample, Article 403 of the Italian Civil Code was activated in 20% of the cases (e.g., 22 out 161 patients). This provision governs the intervention of the Public Authority by means of social services in cases of moral or material abandonment by the parents or when there is a threat to the psycho-physical safety of the child. The goal is to promptly remove minors from the situation of abuse they are experiencing.

Follow-up was activated for 76% of the patients. For 133 out of 161 patients the pathway was successful, while for 20 out 161 patients it resulted in negative outcomes (negative outcomes are understood as a failure to improve the patient’s clinical, psychological, or social condition or the family unit expressing their willingness to disengage from services. In addition to this, negative outcomes are also considered to be those related to the cases taken into charge in the first months of the year 2023 because the timeframe of this study did not give the possibility to understand the outcome in the medium to long period).

#### 3.1.11. Distribution of Socio-Demographic and Abuse-Related Characteristics According to Involvement of the Judicial Authority, Presence of Symptoms, and Activation of Services

The study also considered the distribution of socio-demographic and abuse-related characteristics stratified by involvement of the judicial authority, presence of symptoms, and activation of services. The *p*-value of the chi-square test (or Fisher’s exact test when indicated) was used to test the association between the variables.

The statistical analysis revealed an association between the experience of witnessed violence and the involvement of the judicial authority (*p*-value 0.0013). Among the cases for which a report was made, more than half (56%) were patients who had been victims of witnessed violence. Conversely, among the cases for which no judicial action was taken, only 8% of the patients witnessed violence (Table S1).

As can be seen from Table S1, the place of residence (*p*-value 0.0164), educational level (*p*-value 0.0355), duration of violence (*p*-value 0.0105) and drug abuse as an adult (*p*-value 0.0133) are associated with the onset of symptoms. Specifically, individuals who develop symptoms live less frequently in small towns, experience prolonged violence (95.59%), and there is a lower proportion of persons with drug dependence compared to those who do not show symptoms (42.42% vs. 71.43%).

Furthermore, a longer duration of care was noted in patients who developed symptoms related to the abuse compared to patients who did not develop symptoms (*p*-value 0.0305).

Regarding the activation of services, the analysis showed that it was more frequent in cases where the adults had a psychiatric condition (63.64%, *p*-value 0.0065).

#### 3.1.12. Distribution of Socio-Demographic and Abuse-Related Characteristics According to Advice Sought and Outcomes

The study also focused on the assessment of the distribution of socio-demographic and abuse-related characteristics stratified by positive outcome and advice sought. The *p*-value of the chi-square test (or Fisher’s exact test when indicated) was used to test the association between the variables themselves.

As shown in Table S2, an association between a successful outcome and the sex of the victim was found (*p*-value 0.0296). Specifically, the proportion of females among those with a positive outcome is higher than the proportion of females among those with a negative outcome (52.63% vs. 25%).

For what concerns the request for counselling, the analysis shows that it was mainly for patients under parental care (e.g., not attending educational institutions) (33.96%, *p*-value 0.0379).

#### 3.1.13. Distribution of Socio-Demographic and Abuse-Related Characteristics According to the Types of Violence

The distribution of socio-demographic and abuse-related characteristics stratified by specific types of violence (neglect, severe neglect, and witnessed violence) was analyzed. The *p*-value of the chi-square test was used to evaluate the association between the variables.

As for what concerns neglect (Table S3), an association can be noted between this form of violence and the nationality of the victim (*p*-value 0.0376), the place of residence (*p*-value 0.0317), not being Italian (86%). Moreover, the presence of psychiatric pathology in the adult (*p*-value 0.0003) was positively associated with having suffered from neglect. Specifically, in 62% of neglect cases, the adult was affected by a form of psychological weakness. In contrast, the presence of overt psychiatric pathology in the adult was associated with severe neglect (*p*-value 0.0266).

Furthermore, the analysis shows an association between the level of education and witnessed violence (*p*-value 0.0446).

The duration of care was longer in individuals who suffered severe neglect compared to those who did not (*p*-value 0.0451) and was equally long in children who were subject to witnessed violence (*p*-value 0.0109).

Victims who experienced psychological violence were older compared to those who did not (*p*-value 0.0205). There were no other variables associated with this form of abuse, probably due to the very small number of subjects involved in the study who experienced it (Table S4).

As for what concerns physical abuse, 58% (*p*-value 0.0445) of those who suffered it were not Italian (Table S4).

### 3.2. Hospital Level

Between 2017 and 2023, there were 1586 recorded accesses to the pediatric EDs of Verbania and Domodossola for trauma, falls, burns, and adjustment disorder with anxiety and depressed mood. Of these, 24 accesses resulted in admission to the pediatrics department but only in one case with an overt diagnosis of child maltreatment. The remaining accesses led to discharge but in no case were the “diagnosis code” and the description of discharge diagnosis child-abuse-specific.

### 3.3. Bridging Results Between Territorial and Hospital Level

Since the study aimed also to investigate if a potential pattern of diagnosis existed among victims of child abuse seeking care, previous access to the ED of minors taken in charge by MTA team were thoroughly considered. Patients managed by the MTA team (*n =* 6) had already accessed the ED on several occasions in previous years. For five out of six victims the most frequently detected pattern of diagnosis was “accidental trauma”.

Twelve accesses for the diagnosis “minor trauma” were observed for one patient between 2010 and 2023, with half of them relating to accidental trauma.

Similarly, another patient accessed the ED on nine separate occasions between 2010 and 2023, in five cases due to accidental trauma. When the victim’s mother accessed care in 2023 due to episodes of GBV, the “Codice Rosa” (the “Codice Rosa”, regulated in Piedmont by regional law 4/2016, serves as an access route to the ED reserved for victims of violence and discrimination. The code is visible only to health professionals, and it enables the activation of the multiprofessional team consisting of gynecologists, pediatricians, midwifes, nurses, ED health personnel, forensic doctors, social workers, and other professionals) was activated, and the child was taken into care by the MTA team. However, this access was not reported with a specific “diagnosis code” on the ED’s discharge form of the child who had suffered witnessed violence and therefore was not initially identified. This is due to the fact that “Codice Rosa” is not associated with a parallel “diagnosis code” for the children of victims of GBV. The assignment of this code was discovered later on during the cross-check of data by specifically analyzing case files and medical records in the child neuropsychiatry department.

## 4. Discussion

At the territorial level, the findings of this study indicate that, in the VCO area, violence against minors predominantly manifested as witnessed violence, accounting for approximately 53% of cases. This figure is markedly higher than the national data reported by the “Second National Survey on the Maltreatment of Children and Adolescents in Italy” (32.4%) [8], where neglect and pathology of care were the most frequently identified forms of maltreatment. This discrepancy suggests a significant prevalence of GBV, particularly domestic or intimate partner violence, within the examined area.

The high proportion of witnessed violence cases may be interpreted through a dual lens. On one hand, it may reflect increased awareness among women experiencing violence, leading to higher reporting rates. On the other hand, it highlights a strong sense of responsibility and preparedness among professionals, institutions, and community members in identifying and addressing these situations.

Focusing on the analyzed period, data showed that, in 2019, the year in which the MTA team was established, the number of registered intakes increased compared to the two previous years. This finding underscores the relevance of the Piedmont Regional Council Resolution No. 42-29997, which promoted the implementation of multidisciplinary teams to ensure comprehensive management of child abuse and maltreatment cases [9].

During the COVID-19 pandemic, a decline in intakes was observed in 2020, alongside a partial reduction or interruption of services. A total of 27 cases were reported that year. These findings are consistent with international data reported by UNICEF [10], indicating widespread disruption of child protection services during the pandemic. Importantly, the reduction in reported cases does not suggest a real decrease in abuse incidence. Rather, lockdown measures forced children into prolonged exposure to potentially abusive environments, particularly within households, which accounted for 96% of abuse cases in this study. Fear of retaliation, social isolation, and care-seeking avoidance due to infection risk likely contributed to the emergence of a “hidden” crisis, as described in the literature [11]. In an unsafe environment where the violence may have occurred, even non-abusive family members might have refrained from taking the victim to the ED or reporting the violence for fear of becoming victims themselves. Additionally, limited access to pediatric emergency services may have occurred due to “care-seeking avoidance” by users (e.g., the non-abusive parent who could have accompanied the abused child to the hospital) out of fear of infection. This hypothesis is in adherence with the literature [12].

In contrast, the number of intakes by the MTA Team peaked in 2021 (from 27 in 2020 to 42 in 2021). This surge may be attributed to the cumulative effects of economic instability, job loss, psychological stress, and mental health challenges experienced by families following prolonged restrictions. Phenomena such as parental burnout, combined with the easing of lockdown measures, likely facilitated greater awareness among non-abusive caregivers and enhanced case detection across the territory [13].

In 2023, there were 23 cases of minors accessing the service (the lowest number recorded since the team was set up). This figure can be explained by the fact that the study period for 2023 ended in August rather than at the end of the year, meaning that cases taken on during the last four months of 2023 were not included.

A crucial aspect for the proper care of minors by multidisciplinary teams and consequently for tertiary prevention (e.g., taking care of the child victim of violence with the aim of preventing reoffending and reducing harm through psychotherapy) is early detection, followed by a “qualified reporting” (e.g., a report based on a well-founded suspicion of the alleged fact) [14]. The results of this study revealed a possible previous lack of reporting in 8% of cases. Elements that could have positively impacted early reporting were difficulties experienced by mothers in the postpartum period, in particular postpartum depression, fragile family and social networks with minimal daily life support, fragmented education, including a tendency to drop out of educational programs, and high parental conflict.

Conversely, data regarding the activation of the territorial protection network were highly encouraging. In 96% of cases, the network was promptly engaged, ensuring timely protective responses. Nevertheless, further improvements are warranted, such as the establishment of a dedicated outpatient clinic for abuse and maltreatment, modeled after the “Bambi” clinic at Regina Margherita Pediatric Hospital in Turin. Such an initiative could strengthen continuity of care and reinforce integration between healthcare services and the community [3].

From a preventive perspective, this study emphasizes the importance of shifting the focus towards primary and secondary prevention strategies aimed at enhancing resilience at individual, family, and community levels. In line with the current literature, prevention systems should actively operate within contexts of risk rather than solely intervening after harm has already occurred [15].

Notably, in 72% of cases, abusive or neglectful adults had a personal history characterized by adverse childhood experiences, including exposure to violence, neglect, socio-economic hardship, or complex migration backgrounds. These findings support the hypothesis of transgenerational transmission of violence and “traumatic repetition”, whereby unresolved childhood trauma may contribute to long-term psychiatric vulnerability and impaired caregiving capacities [4,16,17].

This suggests the possibility of a vicious cycle, where past abuse can lead to long-term psychiatric conditions that can negatively impact the ability to care for and meet the primary needs of children.

In addition, psychiatric vulnerability among caregivers emerged as a significant factor. Overt psychiatric disorders were identified in 27% of cases, while an additional 43% of minors exhibited substantial psychological fragility without a formal diagnosis. A strong association was observed between psychological instability and neglect: 62% of neglect cases involved psychologically fragile caregivers, and severe neglect was frequently linked to diagnosed psychiatric conditions. These findings align with existing evidence on the role of maternal postpartum depression and paternal psychological fragility in jeopardizing childcare [18] and paternal psychological fragility [19].

At the hospital level, significant critical issues were identified regarding the detection and documentation of child maltreatment. Analysis of ED revealed that only one admission to the pediatric ward resulted in an explicit diagnosis of abuse, while ED discharge forms lacked any specific diagnostic codes related to neglect or maltreatment.

This lack of standardized diagnostic coding highlights a disconnection between territorial services and hospital management of abuse cases and is consistent with the international literature, which identifies the ED as a high-pressure environment where “time pressure” and a focus on acute clinical stabilization frequently sideline the complex, longitudinal assessment required to identify non-accidental injuries [20,21]. The management of child abuse cases in the VCO area reveals a complex interplay between established protocols and practical institutional constraints. While hospital detection relies on the Italian National Guidelines for the “Codice Rosa”—which focus on acute clinical signs and immediate reporting duties—actual practice often diverges from these frameworks. Factors such as the “primary complaint” focus often mask indicators of witnessed violence, and the lack of standardized, pediatric-specific coding in electronic health records prevents systematic “traceability” [22]. Furthermore, a “defensive” medical approach—driven by the fear of legal repercussions, the perceived administrative burden of the reporting process, or the risk of rupturing the therapeutic alliance with the family—often discourages professionals from documenting suspicions without definitive forensic evidence [23]. These challenges are exacerbated by organizational barriers described in National Guidelines, including high patient volumes, insufficient training, and emotional barriers among staff. The absence of clear documentation not only limited the retrospective evaluation of whether clinical findings were suggestive of abuse versus accidental injury but also prevented meaningful epidemiological comparisons with neighboring areas, such as the province of Novara, highlighting an urgent need for unified recording systems to support child protection frameworks [3,21].

In contrast, territorial management shows a 96% activation rate of the multidisciplinary network. This success could be attributed to specialized teams operating with a “social-health integration” framework, free from the time constraints connected with the management of acute conditions of the ED. This systemic difference allows for a more nuanced assessment of family fragility, explaining why cases not coded as abuse in hospital records are correctly identified once they reach the territorial network. In this context, our findings regarding “patterns of abuse” should be interpreted as epidemiological clinical markers rather than definitive diagnostic proofs. Due to the retrospective nature of the study and the documented “diagnostic silence” in hospital discharge records (where specific codes for abuse were absent), the identified patterns of repeated non-specific trauma represent a “red flag” for missed opportunities in early detection. This underscores the need for standardized regional protocols to bridge the gap between initial ED presentation and confirmed territorial outcomes.

### 4.1. Limitations and Strenghts

Regarding the strengths of the study, it should be noted that this is the first study to analyze the characteristics of cases in the territory under consideration and the management of cases.

One limitation of the study is the data concerning the possible previous lack of reporting, which stands at 8%. In fact, this percentage was determined based on an assessment of practitioners’ familiarity with the significant elements of patients’ stories, clinical peculiarities, and the context in which children were raised. For this reason, these results should be interpreted with caution due to the subjective elements involved. In order to draw more accurate conclusions, other studies should be conducted on a larger sample and employ objective evaluation forms.

It should be noted that one limitation of this retrospective record review is the inherent difficulty in distinguishing between the absence of documentation and the absence of clinical concern or action. As the study relies on existing medical records, cases where clinicians may have had suspicions but failed to document them—or where actions were taken but not recorded—could not be fully differentiated from cases where no clinical concern existed.

On the other hand, the limited results obtained in hospitals in the VCO area were the driving force behind the study itself. This shortcoming, in fact, made it possible to identify some of the critical issues that operators face in managing cases of potential child abuse, identifying, at a practical level, a weak link in the chain relating to the care and management of minors, specifically the moment of discharge.

### 4.2. Recommendations

In the VCO area, the imbalance between the lack of data at the hospital level and the presence of evidence at the territorial level, as highlighted by this study, could encourage policy and operational change. The current study emphasizes the importance of promoting educational and training initiatives that target professionals and focus on the main obstacles encountered in the detection and documentation of child abuse. Furthermore, both within healthcare facilities and in the community, it is essential to treat GBV and violence against minors as public health issues to be prevented, diagnosed, treated, and followed up over time [3].

At the hospital level, considering the ED as the primary point of contact between the patient and the healthcare personnel, specific training is of paramount importance. The findings of the study highlight a significant “weak link” at the discharge stage. Training should thus focus on enabling health professionals to recognize various forms of violence and implement knowledge regarding the legal framework and offenses that can be prosecuted ex officio. A clinical and practical approach through dedicated courses would better support practitioners during complex cases [22]. To fill this gap, a regional coordination group for the clinical and forensic management of abused minors was set up in Piedmont in 2023. It aims to standardize the care pathway of these minors throughout the region (both in hospitals and territorial facilities) and, at the same time, is developing specific training courses for social and health workers involved in the management of these cases.

Clinical practice is often complicated by the difficulty connected with selecting appropriate “diagnosis codes”, a process frequently influenced by medico-legal responsibility factors. This complexity is reflected in the current disconnection found in the results, where no child-abuse-specific codes were identified in ED discharge forms. To bridge this gap, the importance of adopting standardized guidelines that define universal “diagnosis codes” need to be empathized. In the VCO area, this study has already led to practical measures: the Hospital Health Management of Verbania and Domodossola (ASL VCO) has created and shared a dedicated list of “diagnosis codes” with ED staff to ensure these are univocally applied throughout the territory. Furthermore, a specific focus is required for the recognition of witnessed violence, which emerged as the most prevalent form of abuse in the VCO sample, accounting for 53% of cases. The study revealed a critical gap in hospital protocols: when the “Codice Rosa” is activated for a mother victim of GBV, it is often not linked to a specific diagnosis code for the child. This lack of documentation makes the minor’s exposure to violence invisible in hospital records. Therefore, it is crucial to update discharge protocols and standardized guidelines to ensure that witnessed violence is formally and univocally coded, thereby initiating timely territorial protection. Adopting a broader regional and national perspective, it is desirable to create standardized guidelines dedicated to minors that define specific procedures and universal codes. Such guidelines should support the organization of weekly interdisciplinary meetings within facilities to discuss the most challenging cases and develop tailored intake and management pathways. At the regional level, it would be beneficial to support working groups specifically dedicated to minors, rather than only focusing on GBV against adults.

At the territorial level, given that 72% of perpetrators in this study had a personal history characterized by adverse childhood experiences and a high prevalence of psychological fragility (43%) and these were significantly associated during adulthood with neglect towards minors, we recommended implementing targeted primary prevention strategies. Specifically, psychological distress screening programs for pregnant or postpartum parents could be implemented to smooth the transition from hospital discharge to everyday life. These findings suggest that addressing the transgenerational transmission of violence is a key public health priority.

Such programs should also address the prevention of “shaken baby syndrome”. Finally, home visiting programs, especially during the first month of the infant’s life, represent a vital tool for detecting early signs of parental discomfort and domestic violence [23].

## 5. Conclusions

In conclusion, the study conducted in the VCO area allowed for a comprehensive assessment of the local epidemiology of child abuse, revealing critical insights that necessitate a shift in both clinical and territorial management. The new knowledge that emerged from this research lies in the identification of a significant “diagnostic silence” within the hospital setting, contrasted with a high-performing territorial network.

The findings highlight a disproportionately high prevalence of witnessed violence (53%) compared to national averages. This evidence, coupled with the documented gap in hospital diagnostic coding where no child-abuse-specific codes were identified in ED discharge forms, underscores the urgent need to integrate “Codice Rosa” protocols with specific pediatric protective measures. Unlike previous studies focusing solely on clinical signs of abuse, this research highlights how the lack of integrated coding between adult domestic violence and pediatric records leads to the “invisibility” of many minor victims. The consequences for training and documentation systems are centered on the “weak link” identified at the hospital discharge stage. Professional training and standardized diagnostic tools are essential to bridge the current gap between healthcare detection and social protection. Furthermore, the study highlights the importance of maintaining a multidisciplinary team of experts with a shared focus, a model that proved effective in promptly activating the territorial network in 96% of cases.

Finally, priorities for system improvement and future research must address the “transgenerational cycle” of violence, supported by the documented 72% of adverse childhood experiences and 43% psychological fragility among perpetrators. These data provide a novel evidentiary basis for implementing local primary prevention initiatives, such as universal postpartum screening and home-visiting programs, which were previously based on general guidelines rather than local evidence. Future studies should expand this methodology to broader regional areas, using these findings as a baseline to document best practices and map the Italian child abuse management system through objective, data-driven analysis.

## Figures and Tables

**Table 1:** Summary of the kind of violence endured by patients.

	*N* = 161
	*N* (%)
Type of Abuse	
Witnessed violence	85 (52.80)
Neglect	58 (36.02)
Severe neglect	37 (22.98)
Physical abuse	27 (17.77)
Psychological violence	13 (8.07)
Verbal abuse	9 (5.59)
Sexual abuse	8 (4.97)
Peer violence	3 (1.86)
Excessive medical care	2 (1.24)
Extrafamilial witnessed violence	2 (1.24)
**Intrafamily violence**	
No	4 (2.48)
Yes	155 (96.27)
Both	2 (1.24)

**Table 2:** Descriptive statistics on the whole sample concerning unfavorable growing conditions and psychiatric pathology in adult perpetrators.

Characteristics of Adult Perpetrators	*N* = 161
	*N* (%)
**Unfavorable growing conditions**	
No	36 (27.91)
Yes	93 (72.09)
Missing	32
**Psychiatric pathology**	
No symptoms	46 (29.87)
Presence of symptoms	42 (27.27)
Psychological weakness	66 (42.86)
Missing	7

**Table 3:** Distribution of types of violence stratified by presence/absence of involvement of the judicial authority, and *p*-value of the chi-square test, or Fisher’s exact test for the association between type of violence and involvement of the judicial authority.

	Involvement of Judicial Authority		
	No *N* = 12	Yes *N* = 149	
Type of Violence	*N* (%)	*N* (%)	*p*-Value
***Neglect*** **^1^**			
No	6 (50)	97 (65)	0.3532 *
Yes	6 (50.00)	52 (35)	
***Severe neglect*** **^2^**			
No	10 (83)	114 (76)	0.7350 *
Yes	2 (17)	35 (23)	
***Witnessed violence***			
No	11 (92)	65 (44)	0.0013
Yes	1 (8)	84 (56)	
***Physical abuse***			
No	11 (92)	131 (88)	1.000 *
Yes	1 (8)	18 (12)	

^1^ Neglect means the failure of the parent or caregiver to provide for basic needs such as shelter, food, clothing, medical care that, without such, neglect causes physical injury to the child [7]. ^2^ Severe neglect means the failure of the parent or caretaker to provide for basic needs which can result in possible injury or that causes physical harm to the child [7]. * An asterisk indicates *p*-values calculated using Fisher’s exact test.

## Data Availability

The original contributions presented in this study are included in the article/Supplementary Materials. Further inquiries can be directed to the corresponding author.

## Supplementary Materials

The following supporting information can be downloaded at: https://www.mdpi.com/article/10.3390/ijerph23020223/s1, Supplementary File S1: The variables used within the data-collection-form in the territorial setting; Table S1: Distribution of socio-demographic and abuse-related characteristics stratified by involvement of the Judicial Authority, presence of symptoms, and activation of services; Table S2: Distribution of socio-demographic and abuse-related characteristics stratified by positive outcomes, and advice sought; Table S3: Distribution of socio-demographic and abuse-related characteristics stratified by specific types of violence (Neglect, severe neglect, witnessed violence); Table S4: Distribution of socio-demographic and abuse-related characteristics stratified by specific types of violence (phycological violence, and physical abuse).

## Abbreviations

The following abbreviations are used in this manuscript:

WHO	World Health Organization
ED	Emergency Department
ASL	Azienda sanitaria locale
MTA	Multidisciplinary team “Maltreatment, Neglect, Abuse”
GBV	Gender-based violence
VCO	Verbano-Cusio-Ossola
