# Supplementary material for: Intrahospital and Territorial Management of Violence Against Children in the Verbano-Cusio-Ossola Area, Northern Italy
Source: Int J Environ Res Public Health. 2026 Feb 10;23(2):223. doi: 10.3390/ijerph23020223 (PMC12940171; doi:10.3390/ijerph23020223)
Supplement: Supplementary file 1 [file ijerph-23-00223-s001.zip › Supplementary File 1.pdf]

Supplementary file 1

The variables used within the data-collection-form in the territorial setting are 30, and are listed below:

- Year of access to care
- Kind of abuse
- Intra-family/Extra-family violence
- Place where violence occurred
- Protracted event (> 1 month)
- Highlights of the story
- Use of weapons/threats with weapons
- Use of objects during violence
- Gender of the victim
- Age of the patient at the time of care taking
- Country of origin
- Place of birth
- Characteristics of the place of residence
- Education
- Previous failure to report
- Duration of intervention
- Early activation of the local network
- Services sought/Type of services
- Unfavorable growing conditions in adults
- Adult psychiatric pathology
- Drug abuse in adults
- Drug abuse in minors
- Disability

- Symptoms/Type of Symptoms
- Advice sought/Type of advice
- Previous forensic advice sought
- Involvement of the Judicial Autorithy
- Activation of child protection services/ Child protection services
- Follow-up
- Positive outcomes
