# Supplementary material for: Intrahospital and Territorial Management of Violence Against Children in the Verbano-Cusio-Ossola Area, Northern Italy
Source: Int J Environ Res Public Health. 2026 Feb 10;23(2):223. doi: 10.3390/ijerph23020223 (PMC12940171; doi:10.3390/ijerph23020223)
Supplement: Supplementary file 1 [file ijerph-23-00223-s001.zip › Table S1.pdf]

Table S1

Table S1. Distribution of socio-demographic and abuse-related characteristics stratified by involvement of the Judicial Authority, presence of symptoms, and activation of services.

	**Involvement of the Judicial Autorithy**			**Symptoms**			**Activation of child protection services**		
	**No N=12**	**Yes N=149**		**No N=23**	**Yes N=148**		**No N=150**	**Yes N=11**	
	**N (%)**	**N (%)**	**p-value**	**N (%)**	**N (%)**	**p-value**	**N (%)**	**N (%)**	**p-value**
**Sex**									
M	8 (66.67)	72 (48.32)	0.2214	14 (60.87)	66 (47.83)	0.2467	75 (50)	5 (45.45)	0.7710
F	4 (33.33)	77 (51.68)		9 (39.13)	72 (52.17)		75 (50)	6 (54.55)	
**Origin**									
Italian	0 (0)	37 (24.83)	0.0698*	3 (13.04)	34 (24.64)	0.2211	34 (22.67)	3 (27.27)	0.7161*
Not italian	12 (100)	112 (75.17)		20 (86.96)	104 (75.36)		116 (77.33)	8 (72.73)	
**Place of residence**									
Small village (small, tourist, rural)	8 (66.67)	60 (40.27)	0.0520*	16 (69.57)	52 (37.68)	**0.0164**	63 (42)	5 (45.45)	0.5739*
Small town	0 (0)	42 (28.19)		3 (13.04)	39 (28.26)		38 (25.33)	4 (36.36)	
Tourist town	4 (33.33)	47 (31.54)		4 (17.39)	47 (34.06)		49 (32.67)	2 (18.18)	
**Education**									
Infant/nursery school	1 (8.33)	23 (15.54)		4 (17.39)	20 (14.6)		21 (14.09)	3 (27.27)	
Primary school	3 (25)	36 (24.32)		1 (4.35)	38 (27.74)		34 (22.82)	5 (45.45)	
Secondary school	6 (50)	26 (17.57)	0.0744*	4 (17.39)	28 (20.44)	**0.0355***	31 (20.81)	1 (9.09)	0.1354*
High school	2 (16.67)	31 (20.95)		5 (21.74)	28 (20.44)		33 (22.15)	0 (0)	
Parental care	0 (0)	32 (21.62)		9 (39.13)	23 (16.79)		30 (20.13)	2 (18.18)	
Missing	0	1		0	1		1	0	
**Place**									
Home	2 (18.18)	14 (9.4)	0.3025*	2 (8.7)	14 (10.22)	1.0000*	16 (10.74)	0 (0)	0.6040*
Other places	9 (81.82)	135 (90.6)		21 (91.3)	123 (89.78)		133 (89.26)	11 (100)	

Missing	1	0		0	1		1	0	
**Protracted event (>1 month)**									
No	1 (8.33)	10 (6.8)		5 (21.74)	6 (4.41)		10 (6.76)	1 (9.09)	
Yes	11 (91.67)	137 (93.2)	0.5905*	18 (78.26)	130 (95.59)	**0.0105***	138 (93.24)	10 (90.91)	0.5576*
Missing	0	2		0	2		2	0	
**Adult psychiatric pathology**									
No	4 (33.33)	42 (29.58)		6 (27.27)	40 (30.3)		46 (32.17)	0 (0)	
Yes	2 (16.67)	40 (28.17)		3 (13.64)	39 (29.55)		35 (24.48)	7 (63.64)	
Psychological weakness	6 (50)	60 (42.25)	0.7454*	13 (59.09)	53 (40.15)	0.1839	62 (43.36)	4 (36.36)	**0.0065***
Missing	0	7		1	6		7	0	
**Drug abuse in adults**									
No	4 (44.44)	78 (54.17)		6 (28.57)	76 (57.58)		76 (53.52)	6 (54.55)	
Yes	5 (55.56)	66 (45.83)	0.7339*	15 (71.43)	56 (42.42)	**0.0133**	66 (46.48)	5 (45.45)	0.9477
Missing	3	5		2	6		8	0	
**Unfavourable growing conditions in adults**									
No	2 (22.22)	34 (28.33)		8 (42.11)	28 (25.45)		34 (28.57)	2 (20)	
Yes	7 (77.78)	86 (71.67)	1.0000*	11 (57.89)	82 (74.55)	0.1351	85 (71.43)	8 (80)	0.7249*
Missing	3	29		4	28		31	1	
**Previous failure to report**									
No	12 (100)	135 (91.22)		22 (95.65)	125 (91.24)		137 (91.95)	10 (90.91)	
Yes	0 (0)	13 (8.78)	0.6007*	1 (4.35)	12 (8.76)	0.6944*	12 (8.05)	1 (9.09)	1.0000*
Missing	0	1		0	1		1	0	
**Type of abuse**									

Neglect									
No	6 (50)	97 (65.1)	0.3532*	12 (52.17)	91 (65.94)	0.2029	96 (64)	7 (63.64)	1.0000*
Yes	6 (50)	52 (34.9)		11 (47.83)	47 (34.06)		54 (36)	4 (36.36)	
Severe neglect									
No	10 (83.33)	114 (76.51)	0.7350*	20 (86.96)	104 (75.36)	0.2211	117 (78)	7 (63.64)	0.2777
Yes	2 (16.67)	35 (23.49)		3 (13.04)	34 (24.64)		33 (22)	4 (36.36)	
Witnessed violence									
No	11 (91.67)	65 (43.62)	**0.0013**	13 (56.52)	63 (45.65)	0.3337	71 (47.33)	5 (45.45)	0.9041
Yes	1 (8.33)	84 (56.38)		10 (43.48)	75 (54.35)		79 (52.67)	6 (54.55)	
Psychological violence									
No	11 (91.67)	141 (94.63)	0.5112*	23 (100)	129 (93.48)	0.3605*	141 (94)	11 (100)	1.0000*
Yes	1 (8.33)	8 (5.37)		0 (0)	9 (6.52)		9 (6)	0 (0)	
Physical abuse									
No	11 (91.67)	131 (87.92)	1.0000*	21 (91.3)	121 (87.68)	1.0000*	132 (88)	10 (90.91)	1.0000*
Yes	1 (8.33)	18 (12.08)		2 (8.7)	17 (12.32)		18 (12)	1 (9.09)	
	**Median (Q1-Q3)**	**Median (Q1-Q3)**	**p-value^**	**Median (Q1-Q3)**	**Median (Q1-Q3)**	**p-value^**	**Median (Q1-Q3)**	**Median (Q1-Q3)**	**p-value^**
Age	11.5 (8-13)	9 (3-13)	0.2296	3 (2-13)	9 (5-13)	0.0872	9 (4-13)	7 (3-4)	0.0692
Length of taking charge	9 (6.5-15.5)	17 (8-29)	0.0700	9 (6-22)	17.5 (9-30)	**0.0305**	16 (8-27.5)	25 (6-48)	0.4540
