# Supplementary material for: Intrahospital and Territorial Management of Violence Against Children in the Verbano-Cusio-Ossola Area, Northern Italy
Source: Int J Environ Res Public Health. 2026 Feb 10;23(2):223. doi: 10.3390/ijerph23020223 (PMC12940171; doi:10.3390/ijerph23020223)
Supplement: Supplementary file 1 [file ijerph-23-00223-s001.zip › Table S2.pdf]

Table S2

Table S2. Distribution of socio-demographic and abuse-related characteristics stratified by positive outcomes, and advice sought.

	Positive outcomes			Advice sought		
	**No N=20**	**Yes N=133**		**No N=108**	**Yes N=53**	
	**N (%)**	**N (%)**	**p-value**	**N (%)**	**N (%)**	**p-value**
**Sex**						
M	15 (75.00)	63 (47.37)	**0.0296***	54 (50.00)	26 (49.06)	0.9104
F	5 (25.00)	70 (52.63)		54 (50.00)	27 (50.94)	
**Origin**						
Italian	2 (10.00)	31 (23.31)	0.2478*	27 (25.00)	10 (18.87)	0.3848
Not italian	18 (90.00)	102 (76.69)		81 (75.00)	43 (81.13)	
**Place of residence**						
Small village (small, tourist, rural)	6 (30)	57 (42.86)		44 (40.74)	24 (45.28)	
Small town	5 (25)	36 (27.07)	0.3870*	33 (30.56)	9 (16.98)	0.1653
Tourist town	9 (45)	40 (30.08)		31 (28.70)	20 (37.74)	
**Education**						
Infant/nursery school	2 (10)	22 (16.67)		17 (15.89)	7 (13.21)	
Primary school	4 (20)	35 (26.52)		30 (28.04)	9 (16.98)	
Secondary school	2 (10)	27 (20.45)	0.1956*	23 (21.50)	9 (16.98)	**0.0379**
High school	8 (40)	21 (15.91)		23 (21.50)	10 (18.87)	
Parental care	4 (20)	27 (20.45)		14 (13.08)	18 (33.96)	
Missing	0	1		1	0	
**Place**						
Home	2 (10)	13 (9.85)		10 (9.35)	6 (11.32)	
Other places	18 (90)	119 (90.15)	1.0000*	97 (90.65)	47 (88.68)	0.6951
Missing	0	1		1	0	
**Protracted event (>1 month)**						
No	0 (0)	11 (8.4)		7 (6.54)	4 (7.69)	
Yes	20 (100)	120 (91.6)	0.3609*	100 (93.46)	48 (92.31)	0.7506*
Missing	0	2		1	1	
**Adult psychiatric pathology**						

No	5 (25)	40 (31.5)		35 (33.33)	11 (22.45)	
Yes	9 (45)	30 (23.62)	0.1481*	24 (22.86)	18 (36.73)	0.1530
Psychological weakness	6 (30)	57 (44.88)		46 (43.81)	20 (40.82)	
Missing	0	6		3	4	
**Drug abuse in adults**						
No	13 (65)	66 (51.97)		51 (50.00)	31 (60.78)	
Yes	7 (35)	61 (48.03)	0.3387*	51 (50.00)	20 (39.22)	0.2073
Missing	0	6		6	2	
**Unfavorable growing conditions in adults**						
No	3 (16.67)	33 (30.28)		24 (27.91)	12 (27.91)	
Yes	15 (83.33)	76 (69.72)	0.2754*	62 (72.09)	31 (72.09)	1.0000
Missing	2	24		22	10	
**Previous failure to report**						
No	19 (95)	121 (91.67)		99 (91.67)	48 (92.31)	
Yes	1 (5)	11 (8.33)	1.0000*	9 (8.33)	4 (7.69)	1.0000*
Missing	0	1		0	1	
**Type of abuse**						
Neglect						
No	9 (45)	89 (66.92)	0.0791*	70 (64.81)	33 (62.26)	0.7514
Yes	11 (55)	44 (33.08)		38 (35.19)	20 (37.74)	
Severe neglect						
No	15 (75)	101 (75.94)	1.0000*	88 (81.48)	36 (67.92)	0.0547
Yes	5 (25)	32 (24.06)		20 (18.52)	17 (32.08)	
Witnessed violence						
No	13 (65)	58 (43.61)	0.0934*	48 (44.44)	28 (52.83)	0.3165
Yes	7 (35)	75 (56.39)		60 (55.56)	25 (47.17)	
Psychological violence						
No	19 (95)	126 (94.74)	1.0000*	102 (94.44)	50 (94.34)	1.0000*
Yes	1 (5)	7 (5.26)		6 (5.56)	3 (5.66)	
Physical abuse						
No	20 (100)	117 (87.97)	0.1323*	92 (85.19)	50 (94.34)	0.0907
Yes	0 (0)	16 (12.03)		16 (14.81)	3 (5.66)	
	**Median (Q1-Q3)**	**Median (Q1-Q3)**	**p-value^**	**Median (Q1-Q3)**	**Median (Q1-Q3)**	**p-value^**

Age	13 (5.5-14.5)	8 (3-12)	0.0958	9 (5-13)	7 (3-13)	0.1060
Length of taking charge	11 (8-21)	18 (8-30)	0.2438	16 (7-29)	15.5 (9-27)	0.6064
