# Supplementary material for: Intrahospital and Territorial Management of Violence Against Children in the Verbano-Cusio-Ossola Area, Northern Italy
Source: Int J Environ Res Public Health. 2026 Feb 10;23(2):223. doi: 10.3390/ijerph23020223 (PMC12940171; doi:10.3390/ijerph23020223)
Supplement: Supplementary file 1 [file ijerph-23-00223-s001.zip › Table S3.pdf]

Table S3

Table S3. Distribution of socio-demographic and abuse-related characteristics stratified by specific types of violence (Neglect, severe neglect, witnessed violence). *Fisher exact test, ^Wilcoxon Mann-Whitney test

	**Neglect**			**Severe neglect**			**Witnessed violence**		
	**No N=103**	**Yes N=58**		**No N=124**	**Yes N=37**		**No N=76**	**Yes N=85**	
	**N (%)**	**N (%)**	**p-value**	**N (%)**	**N (%)**	**p-value**	**N (%)**	**N (%)**	**p-value**
**Sex**									
M	52 (50.49)	28 (48.28)	0.7878	62 (50.00)	18 (48.65)	0.8853	38 (50.00)	42 (49.41)	0.9406
F	51 (49.51)	30 (51.72)		62 (50.00)	19 (51.35)		38 (50.00)	43 (50.59)	
**Origin**									
Italian	29 (28.16)	8 (13.79)	**0.0376**	28 (22.58)	9 (24.32)	0.8249	17 (22.37)	20 (23.53)	0.8612
Not italian	74 (71.84)	50 (86.21)		96 (77.42)	28 (75.68)		59 (77.63)	65 (76.47)	
**Place of residence**									
Small village (small, tourist, rural)	46 (44.66)	22 (37.93)	**0.0317**	47 (37.90)	21 (56.76)	0.1043	37 (48.68)	31 (36.47)	0.1785
Small town	20 (19.42)	22 (37.93)		36 (29.03)	6 (16.22)		20 (26.32)	22 (25.88)	
Tourist town	37 (35.92)	14 (24.14)		41 (33.06)	10 (27.03)		19 (25)	32 (37.65)	
**Education**									
Infant/nursery school	17 (16.50)	7 (12.28)	0.4683	20 (16.26)	4 (10.81)	0.7686	7 (9.33)	17 (20.00)	**0.0446**

Primary school	28 (27.18)	11 (19.30)		28 (22.76)	11 (29.73)		14 (18.67)	25 (29.41)	
Secondary school	17 (16.50)	15 (26.32)		26 (21.14)	6 (16.22)		15 (20.00)	17 (20.00)	
High school	22 (21.36)	11 (19.30)		24 (19.51)	9 (24.32)		21 (28.00)	12 (14.12)	
Parental care	19 (18.45)	13 (22.81)		25 (20.33)	7 (18.92)		18 (24)	14 (16.47)	
Missing	0	1		1	0		1	0	
**Place**									
Home	9 (8.82)	7 (12.07)		16 (13.01)	0 (0.00)		6 (8.00)	10 (11.76)	
Other places	93 (91.18)	51 (87.93)	0.5107	107 (86.99)	37 (100.00)	**0.0240** *	69 (92.00)	75 (88.24)	0.4283
Missing	1	0		1	0		1	0	
**Protracted event (>1 month)**									
No	7 (6.93)	4 (6.9)		11 (9.02)	0 (0.00)		9 (11.84)	2 (2.41)	
Yes	94 (93.07)	54 (93.1)	1.0000 *	111 (90.98)	37 (100)	0.0688 *	67 (88.16)	81 (97.59)	0.0192
Missing	2	0		2	0		0	2	
**Adult psychiatric pathology**									
No	40 (39.6)	6 (11.32)		34 (28.81)	12 (33.33)		21 (28.77)	25 (30.86)	
Yes	28 (27.72)	14 (26.42)	**0.0003**	27 (22.88)	15 (41.67)	**0.0266**	26 (35.62)	16 (19.75)	0.0708
Psychological weakness	33 (32.67)	33 (62.26)		57 (48.31)	9 (25.00)		26 (35.62)	40 (49.38)	
Missing	2	5		6	1		3	4	
**Drug abuse in adults**									

No	58 (59.18)	24 (43.64)	0.0643	58 (50)	24 (64.86)	0.1144	40 (57.14)	42 (50.60)	0.4190
Yes	40 (40.82)	31 (56.36)		58 (50)	13 (35.14)		30 (42.86)	41 (49.40)	
Missing	5	3		8	0		6	2	
**Unfavourable growing conditions in adults**									
No	28 (33.33)	8 (17.78)	0.0605	29 (30.21)	7 (21.21)	0.3203	13 (22.41)	23 (32.39)	0.2087
Yes	56 (66.67)	37 (82.22)		67 (69.79)	26 (78.79)		45 (77.59)	48 (67.61)	
Missing	19	13		28	4		18	14	
**Previous failure to report**									
No	94 (92.16)	53 (91.38)	1.0000 *	112 (91.06)	35 (94.59)	0.7341 *	67 (88.16)	80 (95.24)	0.1017
Yes	8 (7.84)	5 (8.62)		11 (8.94)	2 (5.41)		9 (11.84)	4 (4.76)	
Missing	1	0		1	0		0	1	
	**Median (Q1-Q3)**	**Median (Q1-Q3)**	**p-value^**	**Median (Q1-Q3)**	**Median (Q1-Q3)**	**p-value^**	**Median (Q1-Q3)**	**Median (Q1-Q3)**	**p-value^**
Age	9 (4-13)	9 (3-13)	0.7037	9 (3-13)	9 (5-13)	0.5152	10 (3-14.5)	9 (4-12)	0.3342
Length of taking charge	19 (8-29)	13.5 (7-26)	0.2007	15 (7-27)	24 (11-34)	**0.0451**	13 (7-24)	19 (10-31)	**0.0109**
