# Supplementary material for: Intrahospital and Territorial Management of Violence Against Children in the Verbano-Cusio-Ossola Area, Northern Italy
Source: Int J Environ Res Public Health. 2026 Feb 10;23(2):223. doi: 10.3390/ijerph23020223 (PMC12940171; doi:10.3390/ijerph23020223)
Supplement: Supplementary file 1 [file ijerph-23-00223-s001.zip › Table S4.pdf]

Table S4

Table S4. Distribution of socio-demographic and abuse-related characteristics stratified by specific types of violence (phycological violence, and physical abuse). * Fisher exact test, ^ Mann-Whitney test

	Psychological violence			Physical abuse		
	No<br>N=152	Yes<br>N=9		No<br>N=142	Yes<br>N=19	
	N (%)	N (%)	p-value	N (%)	N (%)	p-value
**Sex**						
M	76 (50)	4 (44.44)	1.0000*	68 (47.89)	12 (63.16)	0.2112
F	76 (50)	5 (55.56)		74 (52.11)	7 (36.84)	
**Origin**						
Italian	36 (23.68)	1 (11.11)	0.6857*	29 (20.42)	8 (42.11)	**0.0445***
Not italian	116 (76.32)	8 (88.89)		113 (79.58)	11 (57.89)	
**Place of residence**						
Small village (small, tourist, rural)	65 (42.76)	3 (33.33)	0.3144*	59 (41.55)	9 (47.37)	0.5624
Small town	41 (26.97)	1 (11.11)		36 (25.35)	6 (31.58)	
Tourist town	46 (30.26)	5 (55.56)		47 (33.1)	4 (21.05)	
**Education**						
Infant/nursery school	24 (15.89)	0 (0.00)	0.1103*	23 (16.2)	1 (5.56)	0.5163*
Primary school	38 (25.17)	1 (11.11)		36 (25.35)	3 (16.67)	
Secondary school	27 (17.88)	5 (55.56)		28 (19.72)	4 (22.22)	
High school	31 (20.53)	2 (22.22)		27 (19.01)	6 (33.33)	
Parental care	31 (20.53)	1 (11.11)		28 (19.72)	4 (22.22)	
Missing	1	0		0	1	
**Place**						
Home	16 (10.6)	0 (0.00)	0.6003*	15 (10.64)	1 (5.26)	0.6956*
Other places	135 (89.4)	9 (100)		126 (89.36)	18 (94.74)	
Missing	1	0		1	0	
**Protracted event (>1 month)**						
No	11 (7.28)	0 (0.00)	1.0000*	8 (5.71)	3 (15.79)	0.1285*
Yes	140 (92.72)	8 (100)		132 (94.29)	16 (84.21)	
Missing	1	1		2	0	
**Adult psychiatric pathology**						
No	44 (30.34)	2 (22.22)	0.5647*	37 (27.41)	9 (47.37)	0.1642

Yes	38 (26.21)	4 (44.44)		37 (27.41)	5 (26.32)	
Psychological weakness	63 (43.45)	3 (33.33)		61 (45.19)	5 (26.32)	
Missing	7	0		7	0	
**Drug abuse in adults**						
No	75 (51.72)	7 (87.50)		70 (51.85)	12 (66.67)	
Yes	70 (48.28)	1 (12.50)	0.0688*	65 (48.15)	6 (33.33)	0.2365
Missing	7	1		7	1	
**Unfavorable growing conditions in adults**						
No	36 (29.75)	0 (0)		33 (27.97)	3 (27.27)	
Yes	85 (70.25)	8 (100)	0.1049*	85 (72.03)	8 (72.73)	1.0000*
Missing	31	1		24	8	
**Previous failure to report**						
No	141 (92.76)	6 (75.00)		131 (92.91)	16 (84.21)	
Yes	11 (7.24)	2 (25.00)	0.1293*	10 (7.09)	3 (15.79)	0.1875*
Missing	0	1		1	0	
	**Median (Q1-Q3)**	**Median (Q1-Q3)**	**p-value^**	**Median (Q1-Q3)**	**Median (Q1-Q3)**	**p-value^**
Age	8.5 (3-13)	13 (11-14)	**0.0205**	9 (4-13)	12 (2-15)	0.4368
Length of taking charge	16 (8-29)	19 (12-27)	0.7573	17 (9-29)	10.5 (4-25)	0.0749
